# Temporal Trends in the Use of Healthcare Services for Respiratory Infections in the Paediatric Population of Anoia (2017–2024): Primary and Hospital Care

**DOI:** 10.3390/v18060586

**Published:** 2026-05-22

**Authors:** María José Macías Reyes, Josep Vidal-Alaball, Laia Sola Reguant, Anna Ruiz-Comellas

**Affiliations:** 1 Faculty of Medicine, University of Vic-Central University of Catalonia (UVic-UCC), 08500 Vic, Spain; mariajose.macias@uvic.cat (M.J.M.R.); aruiz.cc.ics@gencat.cat (A.R.-C.); 2 PhD Programme in Medicine and Biomedical Sciences, Doctoral School, University of Vic-Central University of Catalonia (UVic-UCC), 08500 Vic, Spain; 3 University Hospital of Igualada, 08700 Igualada, Spain; 4 Health Promotion Research Group in Rural Areas, Catalan Institute of Health, 08272 Sant Fruitós de Bages, Spain; 5 Central Catalonia Research Support Unit, Jordi Gol i Gurina University Foundation for Primary Care Research, 08007 Barcelona, Spain; 6 Sant Joan de Vilatorrada Primary Care Centre, 08250 Sant Joan de Vilatorrada, Spain

**Keywords:** respiratory tract infections, paediatrics, health services, COVID-19, epidemiology

## Abstract

Respiratory infections are among the leading causes of healthcare consultations in paediatric populations. The SARS-CoV-2 pandemic significantly altered both the circulation of respiratory pathogens and the utilisation of healthcare services. This retrospective longitudinal observational study analysed temporal trends in consultations for respiratory infections among children under 15 years of age in the Anoia region between 2017 and 2024. Descriptive analyses and time-series modelling using negative binomial regression were performed. A total of 71,918 consultations were recorded, of which 71.7% occurred in primary care and 28.9% in hospital settings. The mean age of patients was lower in the hospital setting (3.4 years) than in primary care (8.7 years). During the pandemic, consultations decreased by 38% compared with the pre-pandemic period, followed by a rebound in 2022, particularly in hospital care. In the post-pandemic period, hospital consultations remained above pre-pandemic levels, whereas primary care activity tended to stabilise. No increase in bronchiolitis consultations was observed compared with the pre-pandemic period.

## 1. Introduction

Globally, acute respiratory infections (ARIs) are the most common cause of infectious diseases in the paediatric population. They are a frequent reason for hospital admission and, in more severe cases, for infant mortality [1,2].

Since the outbreak of the SARS-CoV-2 pandemic in 2019, it has had a major impact on global public health. To mitigate its transmission, measures were implemented, such as home confinement, the use of face masks, hand hygiene, mobility restrictions and the closure of educational centres. The implementation of these measures helped to limit the spread of the virus, and significantly altered the circulation of other droplet-transmitted respiratory pathogens, with a marked decrease in their incidence being observed [1,3,4].

This decrease in the spread of pathogens, together with the perception of risk associated with healthcare centres, translated into a decrease in demand for medical care [5,6]. Several studies documented this phenomenon, both at hospital level and in primary care [6,7,8,9,10]. For example, Wallis et al. [9] reported a 66.75% reduction in visits to paediatric emergency departments.

After the easing of preventive measures, a progressive increase in the incidence of ARIs was observed, reaching peaks higher than those recorded before the pandemic and with variations in the usual seasonal distribution [2]. This could be explained by the lower immunological exposure of the paediatric population during the pandemic period, with the consequent decrease in immunity against other respiratory viruses [11]. In agreement with this, several studies indicate that viruses such as influenza and respiratory syncytial virus (RSV) recovered or exceeded their historical levels, with increases outside the usual season [1,2,12,13].

In addition to pathogen circulation being affected, hospitalisation rates were also altered during the pandemic. Antoon et al. [6] noted a significant decrease in hospitalisations for paediatric respiratory diseases, with the observed-to-expected ratio declining from 1.13 in the pre-pandemic period to 0.38 during the pandemic. Similarly, a change in the referral criteria from primary care to hospital was observed [14].

Given the complexity of respiratory infections and their considerable geographical variability, this study arises from the need to generate evidence on the dynamics of healthcare service use in a specific area such as the Anoia region. Assessing how the COVID-19 pandemic has altered the epidemiology of these infections and the utilisation of healthcare resources is essential for planning prevention strategies and optimising resources. The aim of the study was to analyse the trends in consultations for respiratory infections in the paediatric population of Anoia in both primary and hospital care between 2017 and 2024, with particular attention to the impact of the COVID-19 pandemic. Specifically, the study sought to describe the number of visits for each respiratory condition (J00–J21, H65–H66, B08, V07) by care setting, to compare the proportion of consultations in primary care and hospital emergency departments before, during, and after the pandemic, and to determine whether post-pandemic consultation figures normalised compared with values prior to 2020.

## 2. Methodology

An observational, longitudinal and retrospective study was designed between 2017 and 2024. The study setting was primary care and hospital care. The study population comprised children under 15 years of age residing in Anoia, numbering around 18,916.

All consultations for respiratory symptoms attended by children under 15 years of age in primary care in Anoia or in the emergency department of the reference hospital, the University Hospital of Igualada, during the period between 1 January 2017 and 31 December 2024 were included. No exclusion criteria were established.

The dependent variables included the main respiratory diagnoses coded according to ICD-10: the common cold (J00), sinusitis (J01), acute pharyngitis (J02), tonsillitis (J03), laryngotracheitis (J04), influenza (J09–11), pneumonia (J12–18), acute bronchitis (J20), bronchiolitis (J21), otitis (H65–66), stomatitis (B08) and COVID-19 (U07). The independent variables were age, sex, consultation date and the care setting. The study period was divided into three phases: pre-pandemic (2017–2019), pandemic (2020–2021) and post-pandemic (2022–2024). The unit of analysis was the consultation rather than the individual patient; therefore, repeated visits by the same patient and referrals from primary care to hospital may have resulted in multiple counts.

Statistical analysis: Categorical data were expressed as percentages and frequencies, numerical data as mean and standard deviation. The chi-square test was used to study the association between categorical variables. To compare means, Student’s *t*-test was used if the variables had a normal distribution, and the Mann–Whitney U test was used if the distribution of the variables was non-normal. The level of statistical significance was 5% and the confidence intervals were 95%. The dependent variable of the model was the mean monthly number of consultations for paediatric respiratory infections, aggregated by calendar month during the period 2017–2024. As independent variables, the epidemiological period (pre-pandemic [2017–2019], pandemic [2020–2021] and post-pandemic [2022–2024]) and time as a continuous variable (month–year) were included to capture the temporal trend. As these were count data and overdispersion was observed, a negative binomial regression model was used. The analyses were performed overall and stratified by healthcare setting (primary and hospital care). Seasonality was assessed through the monthly aggregation of consultations and graphical inspection of the time series.

The statistical analyses were carried out using the statistical software R version 4.2.1.

Data collection and information sources: Primary care data were obtained through the Technical Area of the Central Catalonia Regional Office, and hospital data were obtained from the information systems of Hospital d’Igualada. The data were pseudonymised by the Technical Area of each institution. Data were accessed for research purposes between September and November 2025, and the authors did not have access to information that could identify participants during or after data collection. The dataset supporting the findings of this study is publicly available in Mendeley Data at https://data.mendeley.com/datasets/8bpz4cb77v/1, accessed on 16 April 2026 (https://doi.org/10.17632/8bpz4cb77v.1).

Ethical aspects: Ethical approval was obtained from the Jordi Gol Primary Care Research Institute committee, with code 21/041-P and from the Research Ethics Committee of Bellvitge University Hospital, with reference PR307/23 (CSA PR21/2023).

## 3. Results

A total of 71,918 consultations for respiratory infections between 2017 and 2024 were included in the study, mostly managed in primary care (71.7%), with a smaller proportion treated in hospital emergency departments (28.9%) (Table 1). The population seen at hospital level had a lower mean age (3.4 compared with 8.7 years), with those under 5 years predominating (77.9%), whereas in primary care older children predominated (46.8%).

Consultations for bronchiolitis and pneumonia were more frequent in hospital emergency departments, in contrast with conditions such as the common cold or otitis, which were significantly more frequent in primary care. In relation to COVID-19, we can see that there was a higher proportion of consultations in primary care (5.7% compared with 1.5%).

Regarding temporal trends, we can observe a marked decrease in consultations in 2020 at both levels. After the lifting of the lockdown measures in 2022, consultations for respiratory infections increased significantly. In 2023 and 2024, at hospital level, there were high numbers of consultations compared with the pre-pandemic years, while in primary care the figures were more similar to, and even lower than, pre-pandemic levels.

**Figure 1:**
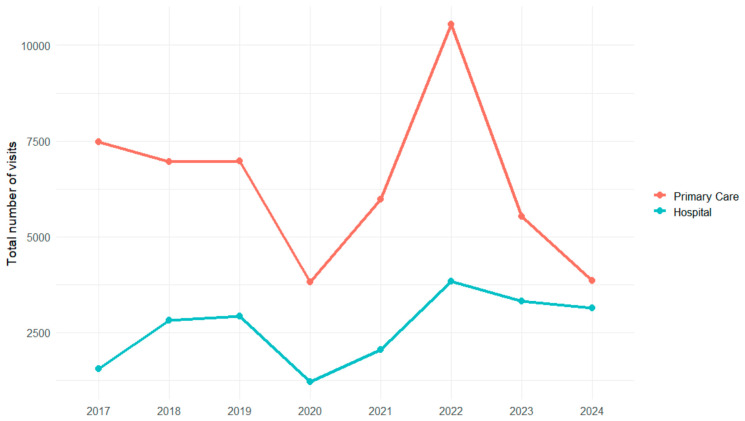
Total number of visits for respiratory infections (2017–2024).

In Figure 1, we can see the abrupt and simultaneous decrease in the total number of visits at both levels of care, more pronounced in 2020, reaching higher figures compared to previous years in 2022, with a downward trend in 2023 but without returning to pre-pandemic values at primary care level. The amplitude of the fluctuations is greater in primary care, while at hospital level, greater stability in demand can be seen.

In Table 2 it can be seen that in the pre-pandemic period the more severe infections, such as pneumonia, bronchitis, laryngotracheitis and bronchiolitis, showed a clear association with the hospital setting, whereas the common cold, pharyngitis, stomatitis or otitis, which normally have a milder course, predominated in primary care; however, tonsillitis showed a similar distribution at both levels (OR close to 1).

During the pandemic, the total number of visits for each respiratory infection decreased both in primary care and in hospital care; there was a higher likelihood of hospital consultation for lower respiratory tract infections. For upper respiratory tract infections, although they remained more frequent in primary care, the relative difference was smaller, which indicates a change in the pattern of consultations with a greater concentration of consultations in hospital.

In the post-pandemic period, a significant increase can be observed in numerous conditions at both levels of care, for example, influenza-like illness, which rose from 467 visits in primary care to 1036, and in hospital from 233 to 590 in absolute numbers. During this period, there was also an association between more severe conditions and hospital care, although with an increase in consultations for conditions that were traditionally managed in primary care; in the case of tonsillitis, the number of visits at hospital level rose from 775 to 1120, and for the common cold from 1151 to 2235 (*p* < 0.001).

It is worth highlighting a marked decrease in consultations for bronchiolitis in the post-pandemic period compared with the pre-pandemic period; the reduction was substantial, particularly at hospital level, falling from 1047 consultations in 2017–2019 to 601 in the years 2022–2024. 

Table 3 provides a descriptive summary of the percentage variation in consultations by diagnosis compared with the mean for the 2017–2019 period. In 2020, substantial decreases were observed across almost all respiratory infections in both primary care and hospital settings. In 2021, a partial recovery was evident, although most diagnoses remained below pre-pandemic levels. In 2022, consultations increased for several respiratory infections, with particularly marked relative increases in influenza-like illness in both care settings. In 2023 and 2024, consultation volumes remained above the pre-pandemic mean for several diagnoses in the hospital setting, whereas in primary care many conditions remained below the average for the 2017–2019 period.

**Figure 2:**
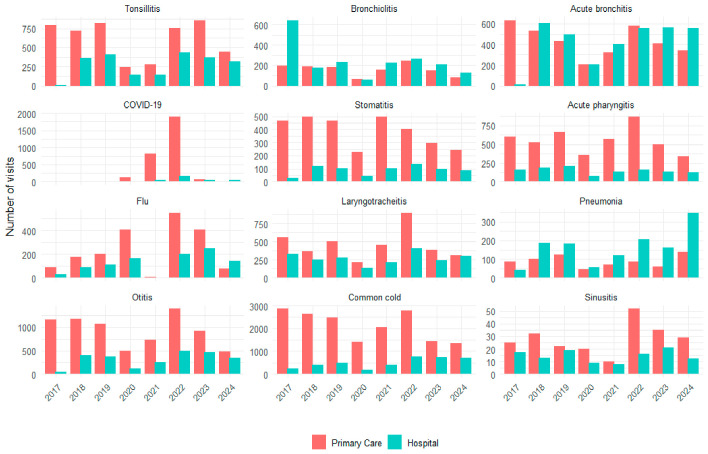
Trend in visits for respiratory infections: comparison between primary and hospital care for each diagnosis.

In Figure 2 we can observe the temporal trend by condition, showing a generalised decline in all infections in 2020, except for influenza-like illness, for which we see figures higher than in previous years, and which was almost nil in 2021.

In Table 4, taking the pre-pandemic period as a reference, an approximate average of 224 consultations per month was estimated. They decreased by 38% with the onset of the pandemic, with a marked decline showing a trend towards significance; no sustained trend was observed in the post-pandemic period, and there was no progressive recovery nor any additional decline.

In Table 5, the reference point is the pandemic; there were an estimated 226 monthly consultations, relating to the period of the lowest use of healthcare services. In the post-pandemic phase, consultations increased strongly and significantly, exceeding pandemic levels.

**Figure 3:**
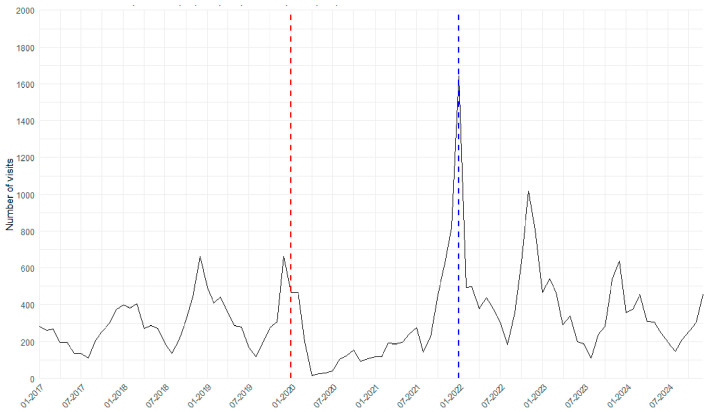
Monthly paediatric consultations for respiratory infections. The dashed lines mark the start of the pandemic (red) and the post-pandemic period (blue).

In Figure 3, we can see a clear seasonal pattern, with relatively stable winter peaks in the pre-pandemic period. When the pandemic began, an abrupt drop was observed, with record lows, and it remained low for several months, without the seasonal variation being significant. Subsequently, a marked recovery was observed in 2022, with a peak higher than in previous winters; in 2023–2024, fluctuations were observed without a sustained linear trend.

## 4. Discussion

The present study identifies four main findings regarding healthcare utilisation for paediatric respiratory infections in the Anoia region between 2017 and 2024. First, a marked decline in consultations was observed in 2020, coinciding with the onset of the COVID-19 pandemic. Second, a significant rebound in healthcare activity was evident in 2022, followed by an irregular subsequent trend. Third, during the post-pandemic period, hospital consultations remained above pre-pandemic levels, whereas in primary care some conditions did not return to pre-pandemic figures. Finally, a decrease in bronchiolitis consultations was observed in the post-pandemic period, particularly in the hospital setting.

This redistribution of healthcare demand is consistent with what was described by Westfall et al. [10]; in primary care, they reported a decrease in consultations for respiratory infections during the pandemic, with a subsequent recovery that was heterogeneous depending on the condition. In the hospital setting, Wallis et al. [9] observed a greater concentration of clinically more significant cases after the pandemic. These results reinforce the existence of a redistribution of healthcare demand, in agreement with what was observed in our study.

Ares-Blanco et al. [15] described that, across Europe, primary care assumed key roles in epidemiological surveillance, contact tracing, diagnostic testing, and the follow-up of patients with COVID-19, while face-to-face consultations declined substantially and telephone and virtual consultations became widely implemented. Similarly, Castillo-Rodenas et al. [16] reported that, during the post-pandemic period, face-to-face paediatric primary care visits recovered, while telemedicine consultations accounted for approximately one quarter of all visits, supporting the emergence of a hybrid model combining in-person and remote care. Therefore, the incomplete recovery of primary care consultations observed in our study, together with hospital consultation volumes that remained above pre-pandemic levels during the post-pandemic period, may reflect not only changes in the circulation of respiratory pathogens, but also system-level adaptations, altered access to primary care, changes in care pathways, and modifications in healthcare-seeking behaviour among families. This interpretation is further supported by Lopes et al. [17] reporting that approximately one in four individuals avoided seeking care, both because of fear of infection in healthcare facilities and because of low confidence in the capacity of health services to manage conditions other than COVID-19. Our study does not allow direct assessment of referral pathways, virtual consultations, or patient decision-making, and this interpretation should therefore be considered exploratory. Our findings regarding the decrease in visits for respiratory infections are consistent with what has been previously described. Antoon et al. [6], Diesner-Treiber et al. [14] and Fakih et al. [18] describe a marked decline in multiple paediatric respiratory illnesses, including bronchiolitis, influenza-like illness, pneumonia, bronchitis and the common cold, with a subsequent increase that, in some cases, exceeded pre-pandemic levels. This pattern is also observed in our study.

The upturn in the number of consultations for respiratory infections observed in our study in the post-pandemic period is consistent with what has been described in various European studies. Falsaperla et al. [19] described an increase in respiratory viruses coinciding with the withdrawal of COVID-19 measures; Staceviciene et al. [2] also described a 27.1% increase in influenza in 2022–2023. These findings support the recovery effect hypothesis following the reduction in viral exposure during the pandemic, similar to the upturn observed in our environment in 2022.

In our study, there was a decrease in consultations for bronchiolitis in the post-pandemic period, especially at the hospital level, in contrast to what has been described in other international studies. Staceviciene et al. [2] and Pun et al. [11] observed increases in RSV with figures comparable to those of the pre-pandemic period. This divergence could be related to local factors, including the introduction of immunisation strategies against RSV in Catalonia from autumn 2023 onwards. However, this interpretation should be considered an exploratory hypothesis, since the observational design of the study and the lack of individual information on vaccination status do not allow us to establish a causal relationship. Specific studies will be required that integrate clinical, microbiological and vaccination coverage data to assess the impact of these interventions on the care burden of bronchiolitis.

As regards the distribution by age groups, our results show a predominance of hospital consultations in children under 5 years of age, while in primary care they were concentrated in older children. This age difference reinforces the clinical vulnerability of the youngest patients and explains their higher hospital attendance. Staceviciene et al. [2] report that between 64% and 82% of cases of COVID-19 and bronchiolitis are concentrated in children under 3 years of age. Similarly, Trempelis et al. [20] described that the clinical impact of SARS-CoV-2 in the paediatric population varied according to the predominant viral variant and the age group affected, with differences observed in both clinical manifestations and patterns of hospitalisation. Consistent with these findings, Wiedenmann et al. [21] reported that infants consistently represented the paediatric subgroup with the highest hospitalisation rates across the different SARS-CoV-2 variants. Taken together, these findings suggest that age-specific susceptibility may have influenced mortality, morbidity, and healthcare utilisation during the pandemic. In contrast, Pun et al. [11] reported that influenza-like illness increases significantly in older children; this is consistent with the higher proportion of consultations in primary care in these age groups.

It is also noteworthy that consultations for COVID-19, both during the pandemic period and in the subsequent years, were more frequent in primary care than at hospital level. Staceviciene et al. [2] describe a decrease in hospitalisations in absolute numbers due to COVID-19 and a lower number of admissions, which supports the argument that the infection generally had a mild presentation in the paediatric population and was mostly managed in outpatient care, in agreement with our results. Population density may also have influenced the transmission dynamics of SARS-CoV-2 observed during the pandemic, as higher population density has been associated with increased incidence after adjustment for demographic, socioeconomic, and environmental factors. Carrión et al. [22] reported that a doubling of population density corresponded to an approximately 6.5% increase in case rates in Catalonia. However, this variable was not specifically assessed in our study.

The percentage variation observed in our study during the pandemic and post-pandemic years, compared with the pre-pandemic years analysed (2017–2019), is consistent with data published internationally, which describe a marked decline in healthcare activity during the pandemic with a subsequent heterogeneous recovery. Antoon et al. [6] specifically described a 91% decrease in bronchiolitis, an 87% decrease in influenza, 81% in pneumonia and 84% in laryngotracheitis. In contrast, Hatoun et al. [23] observed a 99% decrease in influenza during the lockdown period and that in the post-pandemic period it was 85% compared to the pre-pandemic period. These results differ from what we observed in our study, where in 2022 consultations for influenza-like illness almost trebled the pre-COVID values. Finally, for the common cold they report a 70% drop in 2020–2021, subsequently reaching figures similar to those prior to the pandemic, in agreement with our results.

The changes observed between the pre-pandemic and post-pandemic periods were expected, given the impact of the pandemic on the transmission of respiratory pathogens and on healthcare-seeking behaviour. Non-pharmaceutical interventions significantly reduced viral circulation and healthcare demand, while their subsequent relaxation, together with the so-called “immunity debt”, contributed to a resurgence of respiratory infections, with heterogeneous patterns depending on the geographical context. These changes, however, should not be attributed exclusively to non-pharmaceutical interventions. Virus–virus interactions may also have contributed to the altered epidemiological patterns observed during and after the pandemic. Nickbakhsh et al. [24] demonstrated that respiratory viruses can interact at both the population and individual host levels, generating asynchronous patterns of viral circulation. In addition, Maglione et al. [1] showed that the resurgence of paediatric respiratory viruses following the relaxation of COVID-19 restrictions varied substantially according to the pathogen. Together, these findings suggest that the post-pandemic dynamics observed in our study likely reflect the combined effects of public health measures, changes in healthcare-seeking behaviour, immunity debt, and pathogen interference.

## 5. Limitations and Difficulties of the Study

This study has some limitations that should be considered when interpreting the results. Firstly, those inherent to its retrospective design, based on coding by healthcare professionals, which may constitute a source of variability in the recording of diagnoses.

In addition, there is no systematic microbiological confirmation for all the respiratory infections recorded; many are assessed on the basis of symptoms that overlap. However, in certain cases, such as SARS-CoV-2 or influenza, specific tests were carried out. The criteria for screening for these infections varied according to the recommendations at each point in time and the epidemiological context, which may have introduced bias in the identification of cases.

Another important aspect of the study is that the unit of analysis used was the consultation rather than the patient. In this context, the same episode could have been counted on more than one occasion, either due to successive follow-up consultations or referrals from primary care to hospital emergency departments. This circumstance could have led to an overestimation of the absolute volume of healthcare activity due to respiratory infections. However, its impact on temporal trends and comparisons between periods is limited, as the same analysis criterion was applied throughout the entire study period.

Furthermore, changes in access to healthcare and in the criteria for seeking medical attention probably influenced the demand for healthcare, introducing an additional bias.

Lastly, the study is confined to the Anoia area, which limits the generalisation of the findings to other regions with different demographic, epidemiological or healthcare characteristics.

Despite the limitations, the study has strengths, such as the long study period and the inclusion of cases from both primary care and hospital care, providing a broader view of the use of health services in Anoia. We consider that the data obtained constitute an important starting point that could help to guide future research aimed at deepening understanding of patterns of use of health services for respiratory infections in the paediatric population.

## 6. Conclusions

This study describes major changes in the use of healthcare services for paediatric respiratory infections in the Anoia region between 2017 and 2024. An abrupt decrease in consultations was observed in 2020, followed by a subsequent recovery in 2022, which exceeded the pre-pandemic figures, especially at hospital level.

The distribution by age group showed a predominance of hospital consultations among children under 5 years of age, while the 6–10 and 11–15 age groups were concentrated mainly in primary care. No increase in consultations for bronchiolitis was observed in the post-pandemic period. In addition, consultations related to COVID-19 in the paediatric population were managed predominantly in the outpatient setting.

The findings obtained in this study provide important information about patterns of healthcare service use during and after the COVID-19 pandemic and highlight the need to improve systems for monitoring pathogen circulation and planning healthcare resources. Also, the need to improve coordination between primary care and hospital care, in order to respond to future scenarios of epidemiological variability.

## Figures and Tables

**Table 1:** Sample description.

	PC (*n* = 51,097)	Hospital (*n* = 20,821)	*p*-Value
Sex:			<0.001
Female	24,046 (47.1%)	9168 (44.0%)	
Male	27,051 (52.9%)	11,653 (56.0%)	
Median age:	8.67 (3.59)	3.44 (3.64)	0.000
Age:			0.000
0–5 years	10,593 (20.7%)	16,212 (77.9%)	
6–10 years	23,892 (46.8%)	3124 (15.0%)	
11–15 years	16,612 (32.5%)	1485 (7.13%)	
Diagnosis:			
Tonsilitis	4904 (9.60%)	2183 (10.5%)	<0.001
Bronchiolitis	1262 (2.47%)	1927 (9.27%)	<0.001
Acute bronchitis	3460 (6.77%)	3414 (16.4%)	<0.001
COVID-19	2944 (5.76%)	330 (1.59%)	<0.001
Stomatitis	3096 (6.06%)	726 (3.49%)	<0.001
Acute pharyngitis	4413 (8.64%)	1182 (5.69%)	<0.001
Influenza like syndrome	1914 (3.75%)	992 (4.77%)	<0.001
Laryngotracheitis	3677 (7.20%)	2152 (10.4%)	<0.001
Pneumonia	711 (1.39%)	1299 (6.25%)	<0.001
Otitis	7442 (14.6%)	2510 (12.1%)	<0.001
Common cold	17,049 (33.4%)	3950 (19.0%)	<0.001
Sinusitis	225 (0.44%)	115 (0.55%)	<0.001
Year:			0.000
2017	7472 (14.6%)	1557 (7.48%)	
2018	6952 (13.6%)	2812 (13.5%)	
2019	6981 (13.7%)	2918 (14.0%)	
2020	3808 (7.45%)	1209 (5.81%)	
2021	5970 (11.7%)	2040 (9.80%)	
2022	10,540 (20.6%)	3840 (18.4%)	
2023	5526 (10.8%)	3311 (15.9%)	
2024	3848 (7.53%)	3134 (15.1%)	
Period:			<0.001
Pre-pandemic	21,405 (41.9%)	7287 (35.0%)	
Pandemic	9778 (19.1%)	3249 (15.6%)	
Post-pandemic	19,914 (39.0%)	10,285 (49.4%)	

PC: primary care.

**Table 2:** Total number of visits for respiratory infections.

Diagnosis	PRE-PANDEMIC				
	PC (*n* = 21,405)	Hospital (*N* = 7287)	*p*-Value	RD (IC 95%)	OR (IC 95%)
Tonsilitis	2329 (10.88%)	775 (10.64%)	0.575	–0.25% [–1.07%;0.58%]	0.97 [0.89; 1.06]
Bronchiolitis	570 (2.66%)	1047 (14.37%)	<0.001	11.7% [10.8%; 12.5%]	6.13 [5.52; 6.82]
Acute bronchitis	1598 (7.47%)	1117 (15.33%)	<0.001	7.86% [6.95%; 8.76%]	2.24 [2.07; 2.43]
Stomatitis	1430 (6.68%)	254 (3.49%)	<0.001	–3.73% [–3.73%; –2.66%]	0.50 [0.44;0.58]
Acute pharyngitis	1784 (8.33%)	555 (7.62%)	0.056	–0.72% [–1.43%; –0.01%]	0.91 [0.82;1.00]
Influenza like syndrome	467 (2.18%)	233 (3.20%)	<0.001	1.02% [0.57%; 1.46%]	1.48 [1.26; 1.74]
Laryngotracheitis	1427 (6.67%)	855 (11.73%)	<0.001	5.07% [4.26%; 5.88%]	1.86 [1.70;2.03]
Pneumonia	312 (1.46%)	409 (5.61%)	<0.001	4.16% [3.60–4.71%]	4.02 [3.46; 4.67]
Common cold	7984 (37.30%)	1151 (15.80%)	<0.001	–21.5% [–22.5%; –20.5%]	4.02 [3.46; 4.67]
Sinusitis	79 (0.37%)	49 (0.67%)	<0.001	0.30% [0.10%; 0.51%]	1.83 [1.28; 2.61]
Otitis	3425 (16.00%)	833 (11.43%)	<0.001	–4.57% [–5.45%; –3.69%]	0.68 [0.62; 0.73]
COVID-19	-	-	-	-	-
**Diagnosis**	**PANDEMIC**				
	**PC (*n* = 9778)**	**Hospital (*n* = 3249)**	***p*** **-Value**	**RD (IC 95%)**	**OR (IC 95%)**
Tonsilitis	523 (5.35%)	288 (8.86%)	<0.001	3.52% [2.44%; 4.59%]	1.72 [1.48; 2.00]
Bronchiolitis	221 (2.26%)	279 (8.59%)	<0.001	6.33% [5.32%; 7.33%]	4.06 [3.39; 4.87]
Acute bronchitis	526 (5.38%)	609 (18.74%)	<0.001	13.3% [11.9%; 14.7%]	4.06 [3.58; 4.60]
Stomatitis	728 (7.45%)	151 (4.65%)	<0.001	–2.80% [– 3.69%; –1.91%]	0.61 [0.51; 0.73]
Acute pharyngitis	928 (9.49%)	211 (6.49%)	<0.001	–3.00% [–4.02%; –1.97%]	0.66 [0.57; 0.77]
Influenza like syndrome	411 (4.20%)	169 (5.20%)	0.019	1.00% [0.14%; 1.86%]	1.25 [1.04; 1.50]
Laryngotracheitis	658 (6.73%)	353 (10.86%)	<0.001	4.14% [2.96%; 5.32%]	1.69 [1.47; 1.94]
Pneumonia	115 (1.18%)	174 (5.36%)	<0.001	4.18% [3.38%; 4.98%]	4.75 [3.74; 6.04]
Common cold	3462 (35.41%)	564 (17.36%)	<0.001	–18.0% [–19.6%; –16.4%]	0.38 [0.35; 0.42]
Sinusitis	30 (0.31%)	17 (0.52%)	0.107	0.22% [–0.05%; 0.49%]	1.71 [0.94; 3.10]
Otitis	1226 (12.54%)	372 (11.45%)	0.108	–1.09% [–2.37%; 0.19%]	0.90 [0.80; 1.02]
COVID-19	950 (9.72%)	60 (1.85%)	<0.001	–7.87% [–8.62%; –7.12%]	0.17 [0.13; 0.23]
**Diagnosis**	**POST-PANDEMIC**				
	**PC (*n* = 19** **,** **914)**	**Hospital (*n* = 10** **,** **285)**	***p*** **-Value**	**RD (IC 95%)**	**OR (IC 95%)**
Tonsilitis	2052 (10.30%)	1120 (10.89%)	0.121	0.59% [–0.15%; 1.32%]	1.06 [0.98; 1.15]
Bronchiolitis	471 (2.37%)	601 (5.84%)	<0.001	3.48% [2.98–3.98%]	2.56 [2.27; 2.90]
Acute bronchitis	1336 (6.71%)	1688 (16.41%)	<0.001	9.70% [8.91–10.5%]	2.73 [2.53; 2.95]
Stomatitis	938 (4.71%)	321 (3.12%)	<0.001	1.59% [–2.04%; –1.14%]	0.65 [0.57;0.74]
Acute pharyngitis	1701 (8.54%)	416 (4.04%)	<0.001	–4.50% [–5.04%; –3.95%]	0.45 [0.40; 0.50]
Influenza like syndrome	1036 (5.20%)	590 (5.74%)	0.055	0.53% [–0.01%; 1.08%]	1.11 [1.00; 1.23]
Laryngotracheitis	1592 (7.99%)	944 (9.18%)	<0.001	1.18% [0.51%; 1.86%]	1.16 [1.07; 1.27]
Pneumonia	284 (1.43%)	716 (6.96%)	<0.001	5.54% [5.02%; 6.05%]	5.17 [4.50; 5.95]
Common cold	5603 (28.14%)	2235 (21.73%)	<0.001	–6.41% [–7.42%; –5.39%]	0.71 [0.67; 0.75]
Sinusitis	116 (0.58%)	49 (0.48%)	0.270	–0.11% [–0.28%; 0.006%]	0.82 [0.58; 1.14]
Otitis	2791 (14.02%)	1305 (12.69%)	0.002	–1.33% [–2.13%; –0.52%]	0.89 [0.83; 0.96]
COVID-19	1994 (10.01%)	270 (2.63%)	<0.001	–7.39% [–7.91%; –6.87%]	0.24 [0.21; 0.28]

PC: primary care, RD: risk difference. OR: odds ratio. Risk difference: If OR < 1 → more likely in primary care; if OR > 1 → more likely in hospital.

**Table 3:** Variation (in %) in the number of visits for respiratory infections compared with the 2017–2019 mean.

Diagnosis	2020			2021			2022			2023			2024		
	PC	Hospital	Diff AP vs. Hosp (%)	PC	Hospital	Diff AP vs. Hosp (%)	PC	Hospital	Diff AP vs. Hosp (%)	PC	Hospital	Diff AP vs. Hosp (%)	PC	Hospital	Diff AP vs. Hosp (%)
Tonsilitis	–68.6	–43.5	–25.1	–64.1	–45.0	–19.1	–3.0	69.2	–72.2	10.3	42.1	–31.8	–42.9	22.3	–65.2
Bronchiolitis	–65.8	–84.0	18.2	–17.9	–36.1	18.2	27.4	–23.5	50.9	–22.6	–40.1	17.5	–56.8	–64.2	7.4
Acute bronchitis	–61.7	–45.2	–16.5	–39.5	8.8	–48.3	9.1	50.4	–41.3	–23.0	52.3	–75.3	–35.2	50.7	–85.9
Stomatitis	–52.2	–46.9	–5.3	4.9	25.2	–20.3	–15.7	58.3	–74.0	–38.3	19.3	–57.6	–49.2	1.6	–50.8
Acute pharyngitis	–40.5	–58.9	18.4	–3.5	–27.0	23.5	46.3	–13.5	59.8	–17.3	–28.1	10.8	–43.0	–33.5	–9.5
Influenza like syndrome	160.8	115.0	45.8	–96.8	–97.4	0.6	254.0	156.2	97.8	160.2	218.0	–57.8	–48.6	85.4	–134.0
Laryngotracheitis	–56.1	–51.6	–4.5	–5.6	–24.6	19.0	89.0	42.1	46.9	–18.6	–15.4	–3.2	–35.7	4.6	–40.3
Pneumonia	–57.7	–59.7	2.0	–31.7	–12.7	–19.0	–16.3	51.1	–67.4	–43.3	18.1	–61.4	32.7	156.0	–123.3
Common cold	–46.9	–53.3	6.4	–23.0	0.3	–23.3	5.2	101.0	–95.8	–45.5	95.0	–140.5	–49.2	86.6	–135.8
Sinusitis	–24.1	–44.9	20.8	–62.0	–51.0	–11.0	97.5	–2.0	99.5	32.9	28.6	4.3	10.1	–26.5	36.6
Otitis	–56.7	–55.7	–1.0	–35.9	–10.3	–25.6	22.4	79.4	–57.0	–19.5	64.2	–83.7	–58.4	26.4	–84.8
COVID-19	-	-	-	-	-	-	-	-	-	-	-	-	-	-	-

PC: primary care. Diff AP vs. Hosp: difference in % of primary care compared to hospital. Each cell represents the percentage variation in the number of visits compared with the mean of the pre-pandemic period (2017–2019). 0% indicates a volume of visits equivalent to the pre-pandemic mean; +100%, double the number of visits; +200%, treble the number of visits; and −50%, half the number of visits compared to the 2017–2019 mean. Table 3 is intended as a descriptive summary of percentage changes relative to the 2017–2019 mean. No inferential comparisons were performed for these diagnosis-specific percentage changes; therefore, the results should be interpreted with caution, particularly for low-frequency diagnoses where small absolute changes may produce large relative variations.

**Table 4:** Negative binomial regression.

	Estimate	*p*-Value	Rate Ratio	IC 95%
Intercept	5.413	<2 × 10^−16^	224	[149; 349]
Pre pandemic	0.014	0.142	1.014	[0.99; 1.03]
Pandemic	–0.474	0.068	0.622	[0.35; 1.06]
Post pandemic	–0.003	0.779	0.996	[0.97; 1.01]

**Table 5:** Comparison of the pre-pandemic vs. post-pandemic periods.

	Estimate	*p*-Value	Rate Ratio	IC 95%
Intercept	5.420	<2 × 10^−16^	226	[179; 290]
Post pandemic	0.616	<0.001	1.851	[1.35; 2.52]
Pre pandemic	0.274	0.083	1.316	[0.96; 1.79]

## Data Availability

The dataset supporting the findings of this study is publicly available in a data repository: Macías Reyes, María José; Vidal-Alaball, Josep; Solà Reguant, Laia; Ruiz Comellas, Anna (2026), “Tendencias temporales en la utilización de los servicios sanitarios por infecciones respiratorias en la población pediátrica del Anoia (2017–2024): atención primaria y hospitalaria”, Mendeley Data, V1, https://doi.org/10.17632/8bpz4cb77v.1.

## References

[1] Maglione M., Tipo V., Barbieri E., Ragucci R., Ciccarelli A.S., Esposito C., Carangelo L., Giannattasio A. (2025). Changes in Respiratory Viruses’ Activity in Children During the COVID-19 Pandemic: A Systematic Review. J. Clin. Med..

[2] Stacevičienė I., Ivaškevičienė I., Burokienė S., Steponavičienė A., Vaičiūnienė D., Tarutytė G., Jankauskienė A. (2024). Epidemiological changes of acute respiratory infections in children: A single-center experience after COVID-19 lockdown. PLoS ONE.

[3] Treggiari D., Pomari C., Zavarise G., Piubelli C., Formenti F., Perandin F. (2024). Characteristics of Respiratory Syncytial Virus Infections in Children in the Post-COVID Seasons: A Northern Italy Hospital Experience. Viruses.

[4] Mori T., Kitano T., Kitagawa D., Murata M., Onishi M., Hachisuka S., Okubo T., Yamamoto N., Nishikawa H., Onaka M. (2024). Risk of admission requirement among children with respiratory infection in the post-COVID-19 pandemic era. J. Infect. Public Health.

[5] García-García E., Rodríguez-Pérez M., García S.M., Montes R.F., Castañón C.S., Bello M.C.A., Dehli C.R., Pérez-Méndez C., Álvarez M.A.A., Calle-Miguel L. (2022). Change on the Circulation of Respiratory Viruses and Paediatric Healthcare Utilization during the COVID-19 Pandemic in Asturias, Northern Spain. Children.

[6] Antoon J.W., Williams D.J., Thurm C., Bendel-Stenzel M., Spaulding A.B., Ii R.J.T., A Reyes M., Shah S.S., Kenyon C.C., Hersh A.L. (2021). The COVID-19 Pandemic and Changes in Healthcare Utilization for Paediatric Respiratory and Nonrespiratory Illnesses in the United States. J. Hosp. Med..

[7] Iozzi L., Brambilla I., Foiadelli T., Marseglia G.L., Ciprandi G. (2020). Paediatric emergency department visits fell by more than 70% during the COVID-19 lockdown in Northern Italy. Acta Paediatr..

[8] KruKruizinga M.D., Noordzij J.G., van Houten M.A., Wieringa J., Tramper-Stranders G.A., Hira V., Bekhof J., Vet N.J., Driessen G.J.A., van Veen M. (2023). Effect of lockdowns on the epidemiology of paediatric respiratory disease—A retrospective analysis of the 2021 summer epidemic. Pediatr. Pulmonol..

[9] Gómez V.G.W., Apolinario M.H., Santana P.S., Pérez C.H., Rueda N.R., Rubino C.T., Ferrer L.Z., Peña-Quintana L. (2021). Evaluation of changes in paediatric healthcare activity during the COVID-19 state of alarm in the Canary Islands. Public Health Pract..

[10] Westfall J.M., Bonilla A.O., Lapadula M.C., Zingoni P.L., Wong W.C.W., Wensaas K.A., Pace W.D., Silva-Valencia J., Scattini L.F., Ng A.P.P. (2024). Changes in primary care visits for respiratory illness during the COVID-19 pandemic: A multinational study by the International Consortium of Primary Care Big Data Researchers (INTRePID). Front. Med..

[11] Pun J.C.S., Tao K.P., Yam S.L.S., Hon K.L., Chan P.K.S., Li A.M., Chan R.W.Y. (2024). Respiratory Viral Infection Patterns in Hospitalised Children Before and After COVID-19 in Hong Kong. Viruses.

[12] Montes J.B.T., Montes D., Miele A., Baik-Han W., Gulati G., Lew L.Q. (2024). The Impact of COVID-19 Pandemic on Respiratory Syncytial Virus Infection in Children. Pulm. Med..

[13] Pannia P.G., Torres F., Tablado M.R., Ferrero F. (2024). Waiting for the next winter. Outpatient paediatric visits for respiratory infections before, during, and after the COVID-19 pandemic in the city of Buenos Aires. Pediatr. Pulmonol..

[14] Diesner-Treiber S.C., Voitl P., Voitl J.J.M., Langer K., Kuzio U., Riepl A., Patel P., Mühl-Riegler A., Mühl B. (2021). Respiratory Infections in Children During a COVID-19 Pandemic Winter. Front. Pediatr..

[15] Ares-Blanco S., Astier-Peña M.P., Gómez-Bravo R., Fernández-García M., Bueno-Ortiz J.M. (2021). The role of primary care during COVID-19 pandemic: A European overview. Atención Primaria.

[16] Castillo-Rodenas M., Gómez J.Á.V., Fuster-Casanovas A., Catalina Q.M., Vidal-Alaball J., Seguí F.L. (2024). Impact of COVID-19 on the Paediatric Primary Care Model in Catalonia: Analysis of Changes in the Number and Type of Face-to-Face and Remote Visits. JMIR Pediatr. Parent..

[17] Lopes S., Soares P., Gama A., Pedro A.R., Moniz M., Laires P., Goes A.R., Nunes C., Dias S. (2022). Association between perception of COVID-19 risk, confidence in health services and avoidance of emergency department visits: Results from a community-based survey in Portugal. BMJ Open.

[18] Fakih H., Abdulsater N., El Hajj Hussein Z. (2024). Epidemiology of Pediatric Respiratory Tract Infections During the COVID-19 Era: A Retrospective Multicentric Study of Hospitalized Children in Lebanon Between October 2018 and March 2021. Cureus.

[19] Falsaperla R., Sortino V., La Cognata D., Barberi C., Corsello G., Malaventura C., Suppiej A., Collotta A.D., Polizzi A., Grassi P. (2024). Acute Respiratory Tract Infections (ARTIs) in Children after COVID-19-Related Social Distancing: An Epidemiological Study in a Single Center of Southern Italy. Diagnostics.

[20] Trempelis K.P., Kosmeri C., Kalavas P., Ladomenou F., Siomou E., Makis A. (2025). SARS-CoV-2 Variants and Their Impact on Paediatric COVID-19: Clinical Manifestations and Hematological Profiles. Diseases.

[21] Wiedenmann M., Ipekci A.M., Araujo-Chaveron L., Prajapati N., Lam Y.T., Alam M.I., L’HUillier A.G., Zhelyazkov I., Heron L., Low N. (2023). SARS-CoV-2 variants of concern in children and adolescents with COVID-19: A systematic review. BMJ Open.

[22] Carrion-i-Silvestre J.L., García A., López-Bazo E., Moreno R., Ramos R., Royuela V., Suriñach J. (2020). The Effect of Population Density on the Spread of COVID-19 in the Catalan Territory. https://www.ub.edu/aqr_covid19/docs/AQR_Covid19_density_eng.pdf.

[23] Hatoun J., Correa E.T., Vernacchio L. (2022). COVID-19 Pandemic-Related Changes in Paediatric Seasonal Respiratory Infections. Pediatrics.

[24] Nickbakhsh S., Mair C., Matthews L., Reeve R., Johnson P.C.D., Thorburn F., von Wissmann B., Reynolds A., McMenamin J., Gunson R.N. (2019). Virus-virus interactions impact the population dynamics of influenza and the common cold. Proc. Natl. Acad. Sci. USA.

